# May-Thurner Syndrome: An Anatomic Predisposition to Deep Vein Thrombosis

**DOI:** 10.7759/cureus.16682

**Published:** 2021-07-28

**Authors:** Wasey Ali Yadullahi Mir, Dhan B Shrestha, Barun B Aryal, Victoria Lord, Larissa Verda

**Affiliations:** 1 Department of Internal Medicine, Mount Sinai Hospital, Chicago, USA; 2 Department of Emergency Medicine, BP Smriti Hospital, Kathmandu, NPL; 3 Department of Internal Medicine, University of Miami, Palm Beach Gardens, USA

**Keywords:** may-thurner syndrome, deep vein thrombosis, endovascular procedure, iliac vein, iliac artery, mechanical thrombolysis

## Abstract

May-Thurner syndrome (MTS) is a rare clinical condition caused by extrinsic compression of the left common iliac vein by the right common iliac artery, leading to venous stasis and predisposing to thrombus formation. Here, we present the case of a 39-year-old female with no obviously known other risk factors predisposing to thrombosis who presented with severe left leg pain and swelling for a week. The international normalized ratio was elevated and the venous Doppler study showed extensive thrombosis extending from the left common iliac vein to the common femoral vein and the popliteal vein. She was diagnosed with MTS and treated with catheter-directed mechanical thrombolysis and thrombectomy, along with angioplasty of the left common iliac vein and external iliac vein, with near-complete resolution post-treatment.

MTS should be suspected in patients who present with unilateral limb thrombosis regardless of the presence of predisposing factors. Timely management with endovascular procedures is necessary to help prevent other potential life-threatening complications.

## Introduction

May-Thurner syndrome (MTS) is an uncommon anatomical condition caused by extrinsic compression of a vein, most commonly the left common iliac vein, by an artery, most commonly the right common iliac artery, against bony structures, most commonly the fifth vertebral body [1]. This leads to venous stasis and predisposes to the formation of deep vein thrombosis (DVT) [2]. Atypical presentations involving other anatomical structures have also been reported, including right-sided MTS [3-5]. MTS is estimated to be present in 2-5% of cases with lower limb DVT, though the exact prevalence is yet to be established. It is more common in women and the second and third decades of life [6].

Depending on the degree of compression, individuals can be asymptomatic or have venous spur formation with progression to left lower limb DVT [7]. Symptomatic patients may present with acute left extremity swelling and pain, DVT, venous claudication, venous insufficiency, or ulcerations [6-8]. Diagnosis of MTS involves clinical presentation and imaging. While there are no standardized diagnostic criteria, noninvasive ultrasonography is the preferred initial investigation, and the gold standard for diagnosis is conventional venography with intravascular ultrasonography (IVUS). Management includes endovascular interventions, thrombolysis, open surgery, and medical management [2].

Here, we present the case of a 39-year-old female with left lower extremity DVT and pulmonary embolism without any apparent risk factors for thrombosis, who was successfully treated with catheter-directed mechanical thrombolysis and thrombectomy, along with angioplasty of the left common iliac vein and external iliac vein.

## Case presentation

A 39-year-old female presented with severe excruciating left leg pain to gentle touch and left leg swelling. She had been diagnosed and treated conservatively with warfarin elsewhere for extensive proximal left lower extremity DVT and a small right lower lobe pulmonary embolism a week before. The patient was hemodynamically stable but was uncomfortable due to severe pain. Her left leg was almost twice the size of her normal right leg. Physical examination showed pitting edema, focal bluish discoloration over the anterior lower shin, visible varicosities, and peripheral pulses detectable only by Doppler ultrasonography. She had exquisite tenderness to palpation in the left leg and pain with passive motion. However, she was able to move her legs. Her body mass index was 26.8 kg/m^2^.

**Figure 1:**
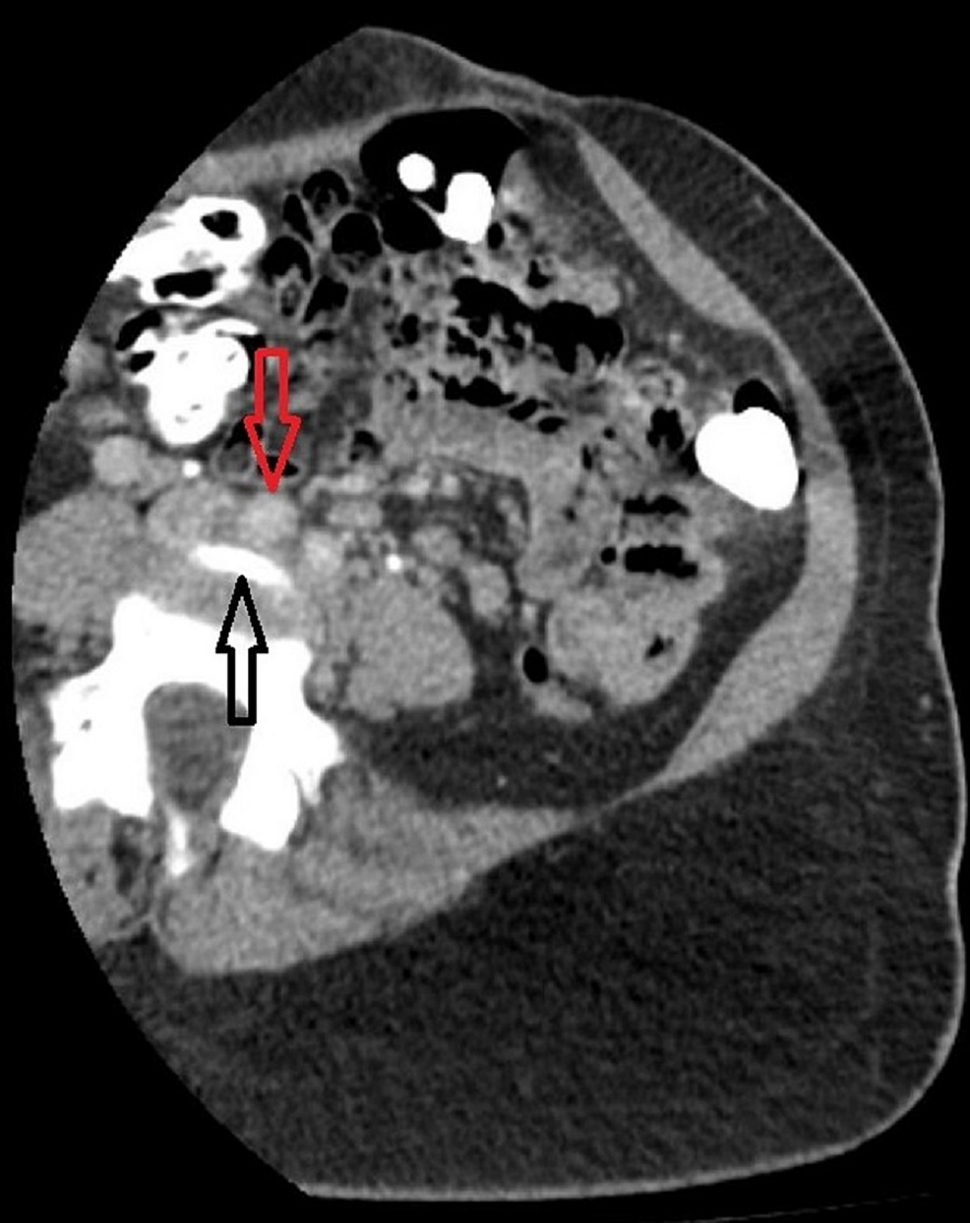
Transverse section of CT pelvis showing compression of the left common iliac vein (black arrow) by the right common Iliac artery (red arrow) in the pelvis. CT: computed tomography

**Figure 2:**
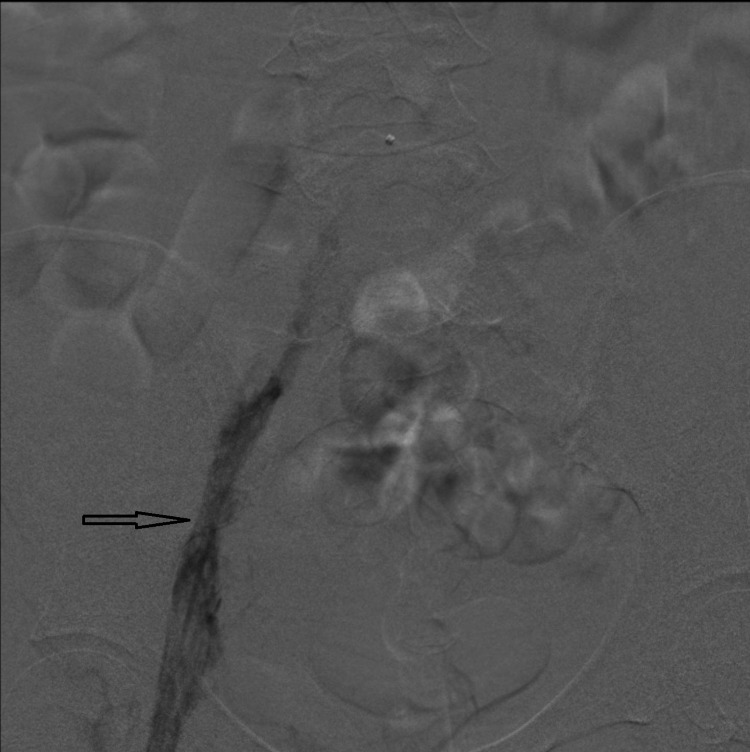
Pre-thrombectomy venogram showing thrombus in the left iliac vein extending to the femoral vein (black arrow).

**Figure 3:**
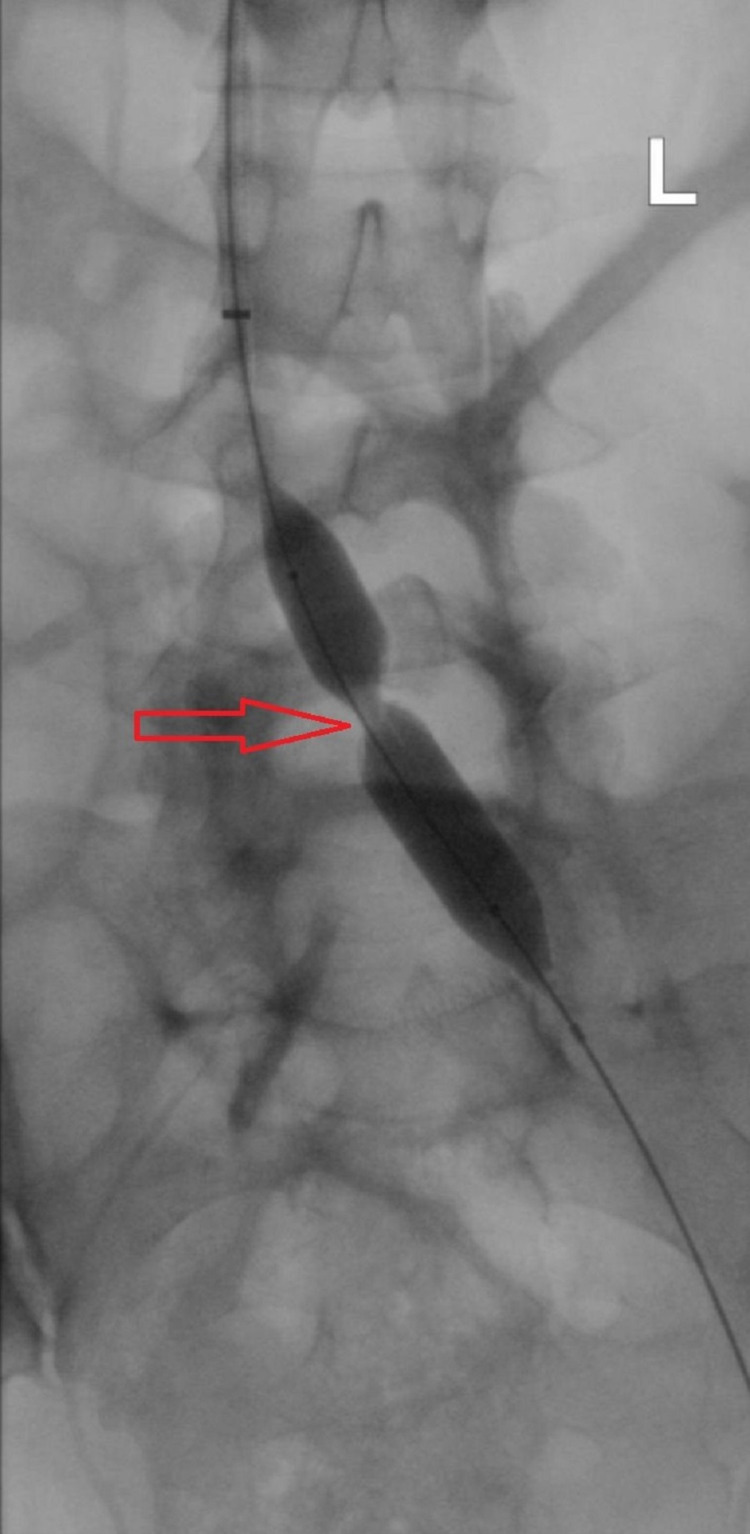
Post-thrombolysis and thrombectomy balloon angioplasty with a waist of the balloon (red arrow) due to external compression by the right iliac artery.

Her past medical history was not significant and she denied any history of smoking or illicit drug use. She was homeless and had a history of two spontaneous miscarriages in the past with no obvious cause, but she denied a history of any medications including oral contraceptive pills. Family history of hypercoagulable states was absent. Laboratory values including renal function tests and lipid profile were unremarkable except for an international normalized ratio (INR) of 1.6. Human immunodeficiency virus and hepatitis C virus serology were negative. Venous Doppler study showed a large clot extending from the left common iliac vein to the common femoral vein and the popliteal vein. CT venogram of the lower extremity confirmed the clot and showed a small amount of thrombus at the inferior vena cava (IVC)/left common iliac vein junction. The above findings suggested the diagnosis of MTS (Figures 1-3).

**Figure 4:**
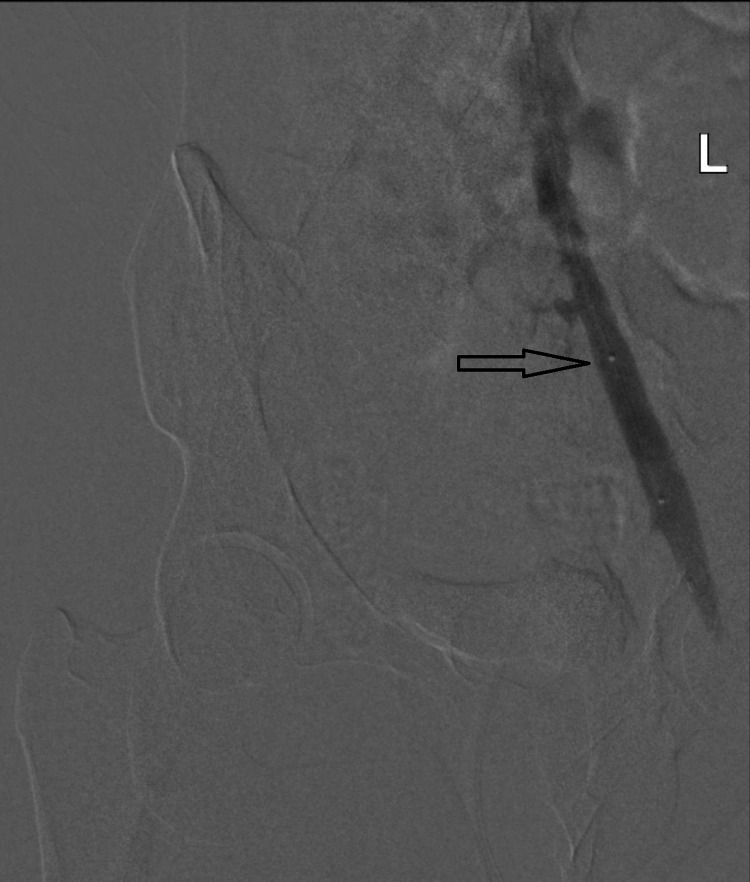
Post-angioplasty venogram (black arrow) showing patent flow and minimal residual stenosis.

**Figure 5:**
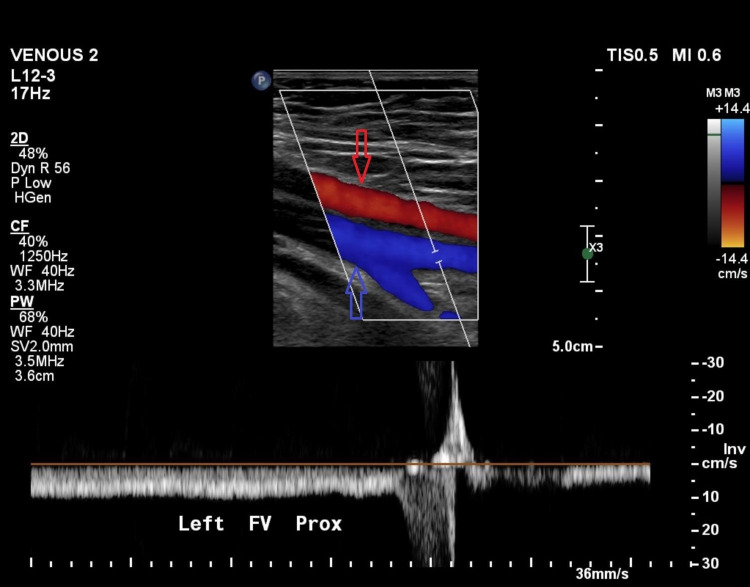
Ultrasound of the pelvis on the six-month follow-up showing patency of the vein (blue arrow) with no residual obstruction, with red arrow showing the overlying artery.

As she was a suitable candidate for mechanical thrombolysis and thrombectomy, she underwent catheter-directed mechanical thrombolysis and thrombectomy of the complete left lower extremity venous system and the left common iliac vein on the third day of admission. She was monitored in the intensive care unit postoperatively and had no complications. On the next day, angioplasty was done for high-grade focal narrowing (>90%) of the left common iliac vein and the external iliac vein, with near-complete resolution post-treatment (Figure 4) and no post-procedure complications. After an additional three days of hospital stay, she has discharged on oral apixaban 10 mg twice a day for one week and outpatient follow-up with a hematologist. After the first week, she was kept on apixaban 5 mg twice a day. One month after discharge, she was readmitted for bleeding and a left mid-foot hematoma, which was evacuated on the bedside. She had no recurrence of thrombosis on six months follow-up (Figure 5). Thereafter, she was lost to follow-up.

## Discussion

DVT is a common and potentially life-threatening condition with an incidence of five per 10,000 individuals per annum in the general population [9]. The frequency of DVT increases with age, with reported incidence rates of 18 per 10,000 individuals per year in the 65-69 age group and 3.1 per 10,000 individuals per annum in the 85-89 age group [10]. It is relatively uncommon in younger age groups before the second decade of life [9,11]. DVT usually occurs in the setting of predisposing factors such as recent surgery or trauma, immobilization of limbs, active cancer, acute medical illnesses, obesity, and thrombophilic disorders.

Venous stasis has been long known to be a predisposing factor for thrombosis and is part of the famous Virchow triad along with hypercoagulability and endothelial injury. One cause of venous stasis was first described in 1957 by May and Thurner, who first observed the formation of a “spur-like formation” in the left common iliac vein where it is crossed by the right common iliac artery [1].

In this report, we presented the case of a 39-year-old female with no apparent risk factors for thrombosis. She presented with massive thrombosis of the left lower extremity and pulmonary embolism. Such a presentation should prompt a search for uncommon causes of DVT. In a young female, MTS is an important but often overlooked possibility. A recent review showed that MTS is twice as common in women, and women also tend to present at a younger age and are more likely to present with pulmonary embolism [2].

Early diagnosis and prompt treatment of thrombosis secondary to external iliac vein compression are essential for acute and chronic management of this condition. In the acute setting, the lower extremity duplex ultrasound may not show DVT if the thrombus is more proximal than expected, which can delay diagnosis and initial treatment. Once DVT is diagnosed, the etiology of iliac venous obstruction may be ascertained by a nonphasic flow in the external iliac vein; however, high common iliac vein velocities are a better indicator of compression [12]. IVUS with conventional venography is the gold-standard modality to diagnose MTS. This can provide accurate sizing of luminal diameter and even provide insights into the chronicity of the thrombus [13]. Reversal of blood flow and formation of collaterals may be visualized.

Treatment of MTS is guided by clinical presentation, mainly the presence or absence of thrombosis. Patients without thrombosis can be managed with thrombolysis and stenting, while management of patients with thrombosis includes thrombolysis, stenting, and angioplasty [6]. Open surgery was widely used in the past but has now been replaced by less invasive treatment options. Endovascular procedures such as catheter-directed thrombolysis, stenting, and IVC filter implantation are the preferred choice for treatment [14,15]. Our patient was treated with catheter-directed mechanical thrombolysis and thrombectomy, followed by angioplasty. She had near-complete resolution of high-grade stenosis post-angioplasty, and additional vascular interventions were not required. After several studies have demonstrated positive results with stenting MTS patients, guidelines from the Society of Interventional Radiology and the Society of Vascular Surgery have been modified to recommend iliac venous stenting in patients who have a compressed external iliac vein [16-18].

## Conclusions

MTS is an anatomical disorder that causes venous stasis in the proximal lower limb and potentially leads to thrombus formation. It should be suspected in young patients, especially women, who present with unexplained DVT in the absence of risk factors for thrombosis. As these cases respond poorly to anticoagulation alone, management with venous angioplasty or stenting, with the removal of thrombus is preferred. Because this is a rare disorder with life-threatening complications such as pulmonary embolism and recurrent thrombosis, a high index of suspicion is warranted for early diagnosis and timely management.
